# Visualized Nucleic Acid Hybridization Lateral Flow Strip Integrating with Microneedle for the Point-of-Care Authentication of *Ophiocordyceps sinensis*

**DOI:** 10.3390/ijms252413599

**Published:** 2024-12-19

**Authors:** Haibin Liu, Xinyue Wang, Hang Tian, Yi Yuan, Jing Wang, Yani Cheng, Linyao Sun, Hongshuo Chen, Xiaoming Song

**Affiliations:** 1 College of Life Sciences, North China University of Science and Technology, Tangshan 063200, China; liuhaibinhappy1013@126.com (H.L.); wangxinyue202409@126.com (X.W.); thtianhang@126.com (H.T.); yuany1459305733@163.com (Y.Y.); blingbling0514@163.com (J.W.); cyn15032577150@126.com (Y.C.); sly18632940311@126.com (L.S.); 2 College of Electrical Engineering, North China University of Science and Technology, Tangshan 063200, China

**Keywords:** double-tailed RPA, *O. sinensis* authentication, lateral flow strip, rapid DNA extraction, nucleic acid hybridization, fast detection

## Abstract

Due to the price and demand of *Ophiocordyceps sinensis* having increased dramatically, adulteration with other fungi is a common problem. Thus, a reliable method of authentic *O. sinensis* identification is essential. In the present work, a rapid DNA extraction and double-tailed recombinase polymerase amplification (RPA) coupled with nucleic acid hybridization lateral flow strip (NAH-LFS) was developed to distinguish authentic *O. sinensis* ingredients from other fungi substitutes. In the presence of *O. sinensis*, the RPA amplicons with two ssDNA tails in the opposite ends, which could simultaneously bind with the SH-probes on gold nanoparticles (AuNPs) and capture the probe on the test line, formed visible red bands. RPA combined with NAH-LFS can efficiently detect *O. sinensis* DNA down to 1.4 ng/μL; meanwhile, the specificity test validated no cross reaction with common adulterants, including *Cordyceps gunnii*, *Cordyceps cicadae*, *Cordyceps militaris*, *yungui Cordyceps*, and *Ophiocordyceps nutans*. The whole RPA-NAH-LFS could be completed within 16 min. The RPA-NAH-LFS results in detecting 20 commercial *O. sinensis* samples are consistent with PCR-AGE and RT-PCR, confirming the feasibility of the RPA-NAH-LFS method. In conclusion, these results are expected to facilitate the application of RPA-NAH-LFS in the authentication detection of *O. sinensis* materials, providing a convenient and efficient method for *O. sinensis* quality control.

## 1. Introduction

*Ophiocordyceps sinensis* (*O. sinensis*), known as “dong chong xia cao” in Chinese, is a traditional Chinese medicinal material that is rarely encountered. Its use as a tonic food and herbal remedy has a history spanning thousands of years, which has contributed to its immense popularity worldwide [1]. *O. sinensis* is a species belong to fungi that includes stromata (family: *Ophiocordycipitaceae*) and shriveled worms (family: *Hepialidae*) as two parts [2,3]. The fungus invades the larva or caterpillar and takes over its body, eventually killing and growing out of the host’s head. *O. sinensis* requires specific environmental conditions to thrive, which are principally found in various high humidity, low oxygen levels, degrees of chilliness, and high-altitude regions (e.g., the snow mountain line or meadows of the Himalayas, Tibetan Plateau, Qinghai, etc.) [4]. As a traditional medicine, *O. sinensis* possesses various medicinal properties, including antioxidant and hypoglycemic properties, boosting the immune system, improving lung and kidney function [5,6]. Due to excessive harvest, habitat destruction, and huge market demand, the price of *O. sinensis* is comparable to gold, and high-quality *O. sinensis* is much more expensive than gold [2]. As a result, *O. sinensis* counterfeit events are becoming increasingly serious, affecting the safe use of *O. sinensis* and causing confusion in the marketplace. Fraudulent producers, lured by the prospect of profit, may substitute authentic *O. sinensis* with various counterfeits that possess a similar morphology, such as *Cordyceps militaris*, *Cordyceps pruinosa*, and *Cordyceps gunnii*, as well as other low-cost fungal species, and even dough, soybean flour, and some undeclared components [7]. However, *O. sinensis* is described as the only authentication recorded in the Chinese Pharmacopoeia. *O. sinensis* is often used as a raw material, but it is also ground into powder and used in health supplements and patented Chinese medicinal products, which makes the authentication of *O. sinensis* impossible based on its morphology. Therefore, it is imperative to establish a simple, cost-effective, and fast quality control method to authenticate *O. sinensis* from counterfeits.

Up to date, numerous analytical methods have been established for *O. sinensis* authentication based on the morphological characteristics, protein, DNA, and other effective components, including polysaccharides and nucleosides. The morphological characteristic-based method, relying on the professional’s subjective judgment and practical experience, has traditionally long been controversial on account of the lack of objective standards [8]. The methods based on protein analysis include enzyme-linked immunosorbent assay (ELISA) [9], ultra-performance liquid chromatography [10], and proteome [11]. Although these methods could effectively authenticate *O. sinensis*, the limitation lies in the reliance on the correct apparatus, technicians, and the presence of denatured proteins which are likely to go undetected in liquid health-care products under complicated processing conditions [10,12]. Among the polysaccharide- and nucleoside-based methods, high-performance liquid chromatography (HPLC), thin layer chromatography (TLC), and liquid chromatography–mass spectrometric detection (LC/MS) are outstanding and widely accepted due to their reliability. However, these methods can only detect a limited number of nucleosides with a relatively high content and are unlikely to enable the detection of trace nucleosides [13]. Furthermore, the content of nucleosides is susceptible to a variety of factors, such as different geographical sources, maturity, and storage. The major drawback of the above methods is that they require sophisticated and expensive instruments and a suitably equipped laboratory. At present, the detection of *O. sinensis* primarily relies on DNA-based assays. DNA exists in all cells of *O. sinensis* and is more conserved than other components. Furthermore, DNA exhibits better thermal stability and is more suitable for processed *O. sinensis* identification [14]. Sequencing, polymerase chain reaction (PCR), and its derived methods like quantitative PCR and digital PCR are usually used in the identification of *O. sinensis* product adulteration [15]. PCR-based methods have the characteristics of high sensitivity and specificity. Nevertheless, PCR-based methods are time-consuming, labor-intensive, and involve lengthy processing times, in addition to requiring thermal cycling equipment. These limitations restrict the application of PCR assays at the point-of-care in the field [16,17].

To avoid the use of thermal-cycling equipment, a variety of isothermal amplification methods were established, such as loop-mediated isothermal amplification (LAMP), recombinase polymerase amplification (RPA), and helicase-dependent amplification (HDA) [18]. RPA is the most widely used technology and has made rapid advancements in recent years. RPA is based on three enzymes (recombinant enzyme, polymerase, and single chain-binding protein) and a pair of primers to realize the amplification of DNA to detectable levels at a lower temperature (37–42 °C) within 20 min [19]. RPA is able to tolerate the impure samples which may inhibit PCR reactions and is available in a lyophilized form, facilitating transportation without requiring cold chain storage [20]. As a whole, RPA is an efficient amplification technique possessing many merits such as high specificity and sensitivity, short turnaround time, and constant incubation temperature [21,22]. However, the RPA amplicons are commonly analyzed using agarose gel electrophoresis (AGE) or fluorescence dye which is time-consuming and exposes analysts to toxic dyes.

Lateral flow strip (LFS) is a portable field diagnostic device, which when used to detect RPA and other isothermal or thermostatic amplification products has many merits, e.g., it is fast, sensitive, convenient, and cheap, and professional technicians and large equipment are not required. The testing results can be observed with naked eyes [23,24]. Combining RPA with LFS (RPA-LFS) is a promising approach because the advantages of both are amplified [25]. In previous LFS research, numerous lateral flow systems (LFSs) have been utilized for the detection of target amplicons by employing specific antigen–antibody binding. This involves modifying antigens to label primers and binding amplicons to gold nanoparticles (AuNPs) that are then captured by the antibodies on LFS. Furthermore, antigens and antibodies are expensive and unstable [26]. These shortcomings render conventional immunoassay LFS costly and time-consuming, thereby hindering their widespread adoption. Recently, nucleic acid hybridization LFS (NAH-LFS) has emerged as a popular alternative to immunological LFS. Apart from inheriting the benefits of traditional LFS, it is also characterized by its affordability, stability, and ease of storage.

In this study, a convenient detection platform was developed for authenticating *O. sinensis* by integrating rapid DNA extraction and double-tailed RPA with NAH-LFS for signal readout. This novel assay features advances over the previously reported methods. First, DNA from *O. sinensis* is extracted using a tungsten needle within just one minute, offering a quick and straightforward process that reduces dependence on specialized equipment and lowers costs. Second, we replaced the traditional antigen–antibody system with single-stranded oligonucleotides and tailing primers. The amplification primers are modified with ssDNA to enhance the binding of amplification products to nanoparticle-labeled reporter probes and capture probes on the lateral flow strip, utilizing base complementarity for precise binding. The optimal specific RPA primers, designed based on the highly conserved mitochondrial gene (RefSeq: KF696589.1) in *O. sinensis*, include a tag sequence and a specific binding region connected by spacer 9 (oxyethylene glycol monomers), which functions to inhibit further primer extension. RPA amplicons with two ssDNA tails bind to sulfhydryl-modified (SH) probes on gold nanoparticles (AuNPs) and capture probes immobilized on the test area (T line), generating visible red bands, while excess AuNPs-SH probes indicate the test’s validity in the control zone. We optimized the double-tailed RPA system and evaluated its sensitivity and specificity. The feasibility of the RPA-NAH-LFS method for *O. sinensis* authentication was tested using commercial *O. sinensis* and related products. This innovative detection tool offers numerous advantages, including low cost, portability, high efficiency, sensitivity, and specificity, making it an excellent choice for point-of-care *O. sinensis* authentication.

## 2. Results

### 2.1. Evaluation of Extracted DNA by Commercial Kit and Tungsten

**Figure 1:**
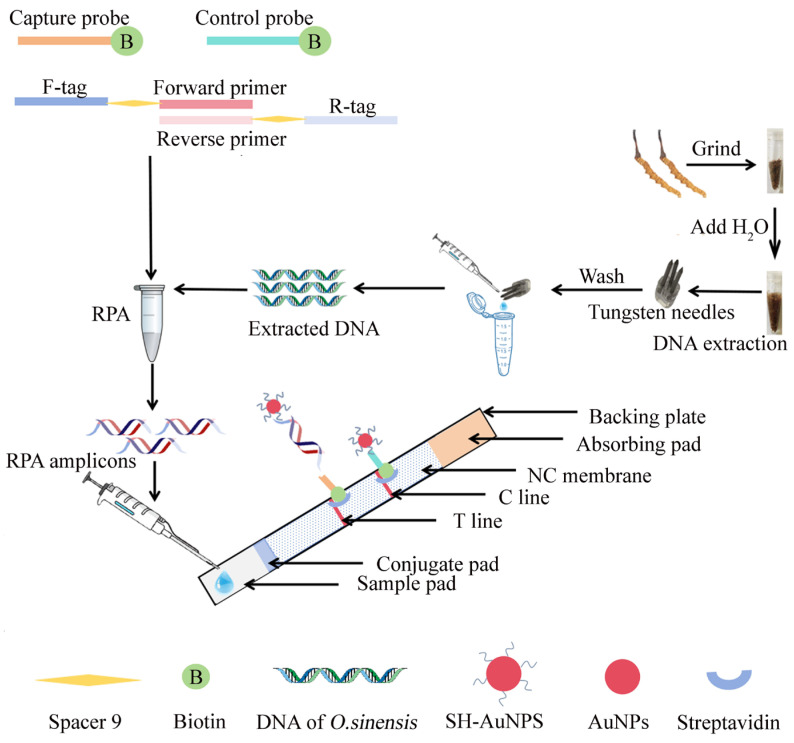
Scheme of the RPA-NAH-LFS for *O. sinensis* authentication detection. F-tag; forward primer; reverse primer; R-tag; control probe; capture probe in different colors means different nucleic acid sequence.

**Figure 2:**
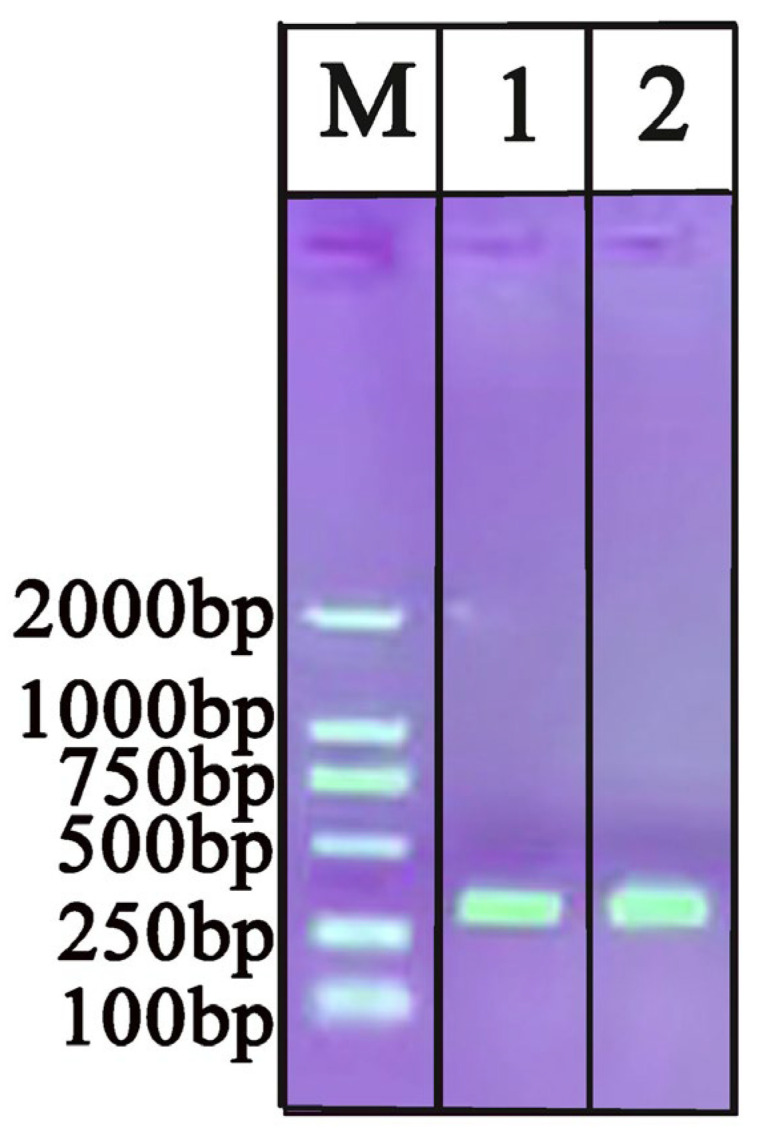
Evaluation of extracted DNA by commercial kit and tungsten through PCR-AGE. 1: DNA extraction with commercial kit; 2: DNA extraction with tungsten; M: D2000 DNA marker.

The process of DNA extraction using a tungsten needle is shown in Figure 1. The capacity of the tungsten needles and the commercial kit for DNA extraction was evaluated by comparing the absorbance ratio (A260/280 and A260/230) of nucleic acids, protein, and other organic contaminations (carbohydrates, EDTA, phenol); the results are shown in Table 1. The DNA extracted using a commercial kit exhibited a high concentration; however, the process was significantly more time-consuming and cumbersome compared to the tungsten method. Both the DNA extracted using tungsten and that from the commercial kit were subjected to PCR-AGE to compare the extraction efficiency. As shown in Figure 2, the PCR-AGE results verify that both the tungsten and commercial kit can obtain good DNA extraction effect.

### 2.2. Characterization of AuNPs and Verification the Formation of AuNPs-SH Probes

**Figure 3:**
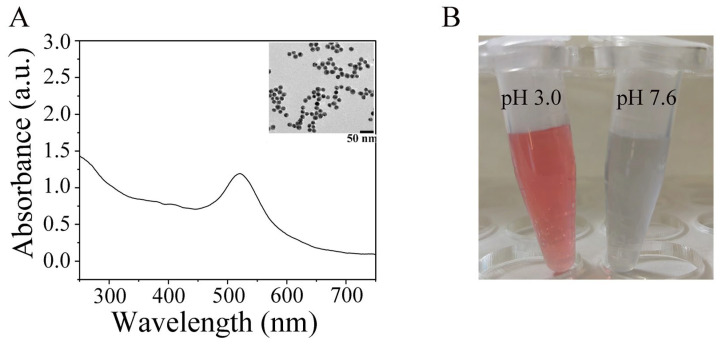
Characterization of AuNPs. (**A**) UV–visible spectrophotometry of AuNPs. Inset shows a TEM image of AuNPs. (**B**) Photographs of AuNP–SH probe solutions mixed with 0.3 M NaCl at pH of 3.0 and pH of 7.6.

The obtained AuNPs were characterized by transmission electron microscope (TEM) and UV-Vis spectrophotometry (Figure 3A). The UV-Vis spectrophotometry showed that AuNPs exhibited an absorption maximum at around 520 nm. The inset shows a TEM image of the AuNPs, with the size of AuNPs being approximately 20 nm. The AuNPs synthesized in this study were burgundy in color. To verify the effect of pH on the coupling of AuNPs and SH probes, the conjunction of the SH probe with AuNPs was tested with the addition of 0.3 M NaCl. As shown in Figure 3B, the SH probes coupled with AuNPs at pH of 7.6 immediately turns colorless, indicating the failure conjugation of AuNPs and SH probes. When the pH is adjusted to 3.0, the SH probes and AuNPs conjunction remain burgundy with the 0.3 M NaCl addition, meaning the successful modification of AuNPs with the SH probes.

### 2.3. Primer Design and NAH-LFS Validation

**Figure 4:**
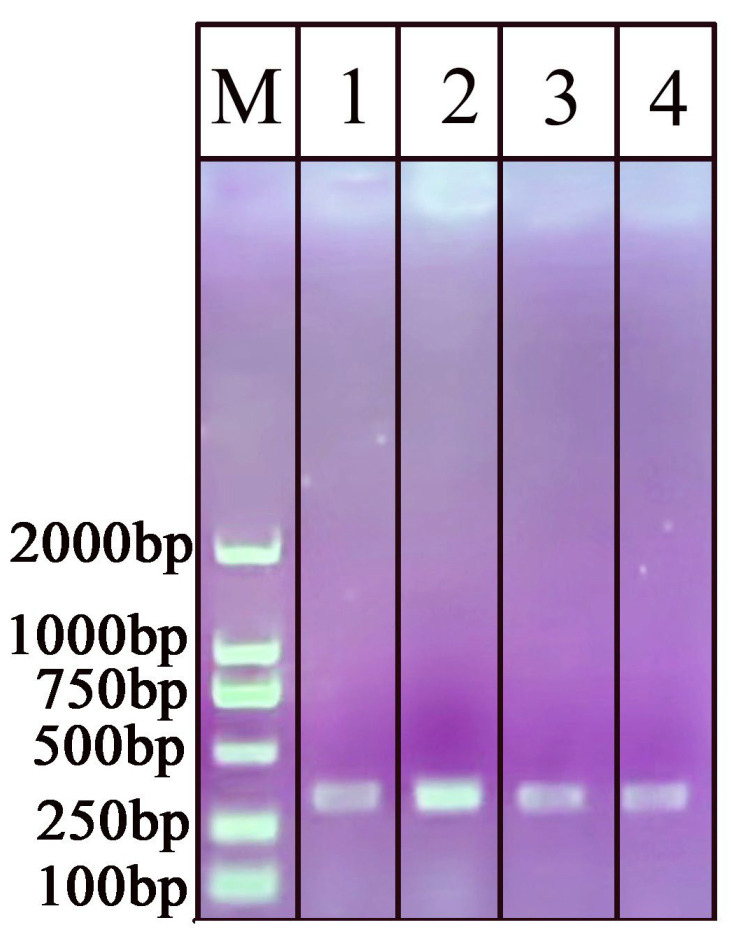
Select the optimum primers pair for *O. sinensis*. M: D2000 DNA marker, lanes 1, 2, 3, and 4 are RPA products with primer 1, 2, 3, and 4 (Table 2), respectively.

Four pairs of primers (1, 2, 3, 4) (Table 2) were designed as candidates and subjected to RPA amplification using the genomic DNA of the *O. sinensis* as template. As expected, primer 2 produced a DNA band of 319 bp that was brighter than the bands generated by the other three primer sets (Figure 4). Therefore, primer 2 was selected as the appropriate primer pair for the amplification of *O. sinensis*. Then, the primer 2 was modified with spacer9 and tag sequence containing the following three functional regions: the first part of the forward/reverse primers must participate in the RPA amplicon located at the 3′ end of the primer, namely the specific binding region; the second part is a spacer region tag (spacer9) as a link to stop DNA polymerase amplification; the third part is a characteristic tag sequence located at the 5′ end of the primer that is maintained as single-stranded during amplification. The RPA products generated with the modified primer2 and *O. sinensis* DNA as the template resulted in the NAH-LFS displaying two red bands at both the C line and T line. In contrast, the negative control with double distilled H_2_O (ddH_2_O) as the template only showed a single red line at the control line, confirming a successful and valid test run of the NAH-LFS assay.

### 2.4. Optimization of RPA and the NAH-LFS Stability Under Room Temperature

**Figure 5:**
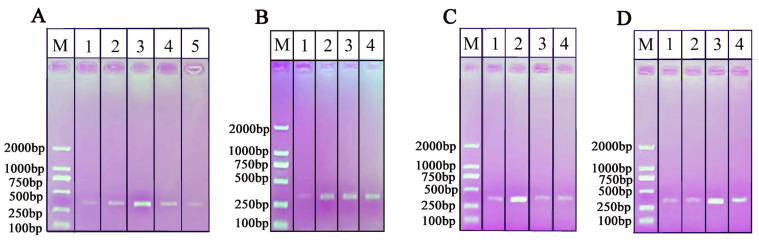
Optimization of RPA conditions. (**A**) Incubation temperature. 1: 37 °C; 2: 38 °C; 3: 39 °C; 4: 40 °C; 5: 41 °C. (**B**) Incubation time. 1: 5 min; 2: 10 min; 3: 15 min; 4: 20 min. (**C**) The concentration of Mg^2+^. 1: 0.56 μM; 2: 0.7 μM; 3: 0.84 μM; 4: 0.98 μM. (**D**) The concentration of primers (10 μM). 1: 0.01 nM; 2: 0.012 nM; 3: 0.014 nM; 4: 0.016 nM. M: D2000 DNA marker.

To enhance the performance of RPA-NAH-LFS, it is essential to explore and optimize the crucial parameters. As the accuracy of RPA is crucial for the subsequent NAH-LFS, RPA amplifications were conducted under varying conditions, including different incubation times and temperatures, as well as the concentrations of Mg^2+^ and primer concentrations. As shown in Figure 5A, the incubation temperatures ranging from 38 °C to 40 °C produced single bands in AGE, with the optimal amplification temperature observed at 39 °C. In Figure 5B, the band brightness increased with the incubation time and stabilized at 10 min, as observed with the naked eyes. To streamline the process, a final incubation time of 10 min was selected. Figure 5C,D illustrate the optimization of primer and Mg^2+^ concentrations, revealing that Mg^2+^ concentrations of 2.5 μL (280 mM) and primer concentration of 1.8 μL (10 μM) produced the clearest bands. Therefore, the optimal RPA reaction conditions include an incubation temperature of 39 °C, an incubation time of 10 min, a Mg^2+^ amount of 2.5 μL (280 mM), and a primer amount of 1.8 μL (10 μM).

**Figure 6:**
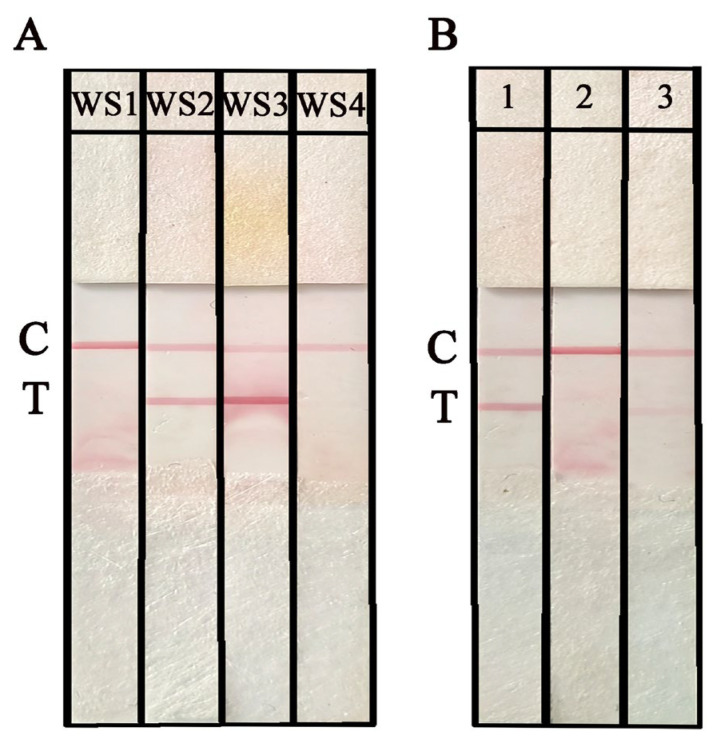
Optimization of NAH-LFS working conditions. (**A**) Optimization of WS. WS1: 0.01 M PBS, pH of 7.0; WS2: 0.01 M PBS with 0.05% Tween-20 and 1% BSA, pH of 7.0; WS3: 0.01 M PBS, pH of 8.0; WS4: 0.01 M PBS, 0.05% Tween-20 and 1% BSA, pH of 8.0. (**B**) Optimization the type of NC membranes. 1: FR = 135 s/4 cm; 2: FR = 180 s/4 cm; 3: 90 s/4 cm. C: control line, T: test line.

**Figure 7:**
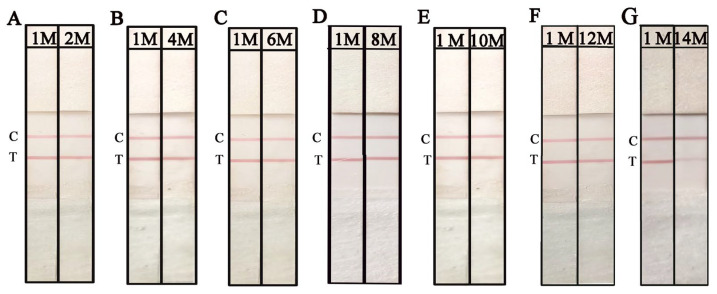
The retention period of NAH-LFS for 2 months (**A**), 4 months (**B**), 6 months (**C**), 8 months (**D**), 10 months (**E**), 12 months (**F**), and 14 months (**G**). 1 M, 2 M, 4 M, 6 M, 8 M, 10 M means NAH-LFS stored for 1 month, 2 months, 4 months, 6 months, 8 months, 10 months, respectively. C: control line; T: test line.

The working solution (WS) of NAH-LFS is another critical factor that can impact the sensitivity and reproducibility of NAH-LFS. In this study, WS1, WS2, WS3, and WS4 were employed to determine the optimal WS. From the results shown in Figure 6A, it is evident that WS2 yielded the best visual outcomes in terms of T/C line color intensity, distribution, and minimal background effects. Based on these findings, WS2 was used in subsequent experiments. Additionally, different types of NC membranes with varying pore sizes were evaluated. As depicted in Figure 6B, the flow rate (FR) of NC membrane on strip 1 (FR = 135 s/4 cm) demonstrated a superior performance as it facilitated the migration of RPA amplicons-AuNPs effectively. Therefore, the NC membrane (FR = 135 s/4 cm) was used for the subsequent experiments. RPA amplification using *O. sinensis* DNA (approximately 50 ng/μL) as template was subjected to NAH-LFS, which was stored for different months to evaluate the retention period. As shown in Figure 7 and Figure S1, the NAH-LFS can be stored at room temperature with a silica desiccant for up to 12 months without significant loss of activity. After more than 12 months, the color of the strips began to lighten. 

### 2.5. Specificity of the RPA-NAH-LFS Assay

**Figure 8:**
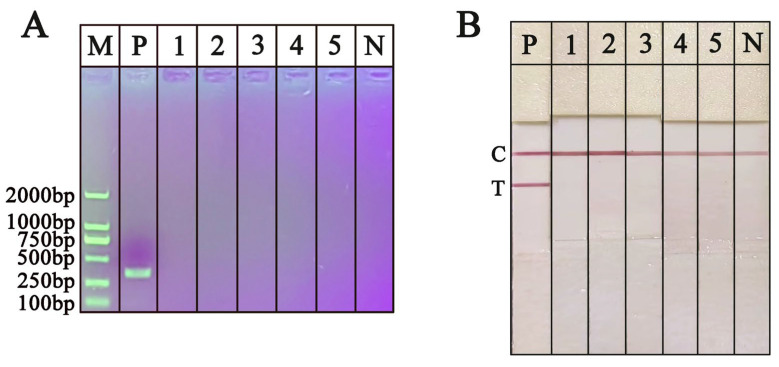
Specificity detection of RPA-AGE (**A**) and RPA-NAH-LFS (**B**). P: *O. sinensis*; 1: *C. gunnii*; 2: *C. cicadae*; 3: *C. militaris*; 4: *O. nutans*; 5: *yungui Cordyceps*; M: D2000 DNA marker; N: negative control; C: control line; T: test line.

To evaluate the specificity of the RPA-NAH-LFS, DNA extracted from *O. sinensis* and other fungal species using a tungsten needle was utilized as the PCR or RPA template. The PCR and RPA products were subsequently analyzed using AGE and NAH-LFS, respectively. As shown in Figure 8A, only the DNA from *O. sinensis* produced a bright band at the expected size of 319 bp by PCR-AGE, while DNA from the five non-target fungal species did not show any bands. In addition, as shown in Figure 8B, RPA products with *O. sinensis* DNA as the template produced clear red bands at both the T and C lines of the NAH-LFS, indicating the presence of *O. sinensis*. In contrast, the other five fungal species exhibited red band only at the C line, indicating the absence of *O. sinensis*. The results obtained from both PCR-AGE and RPA-NAH-LFS are consistent, demonstrating that the developed RPA-NAH-LFS assay exhibits high specificity and shows no interaction with other non-target fungal species.

### 2.6. Sensitivity of the RPA-NAH-LFS Assay

**Figure 9:**
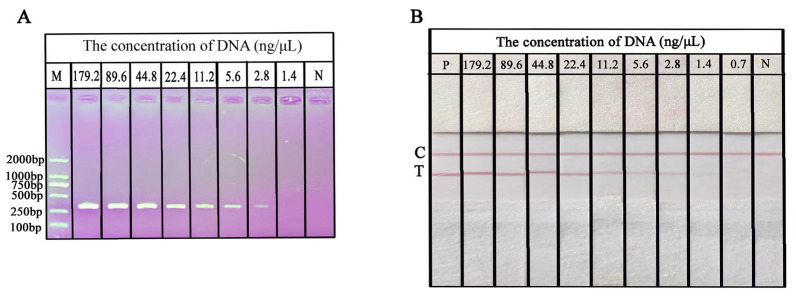
Sensitivity detection of PCR-AGE (**A**) and RPA-NAH-LFS (**B**). M: D2000 DNA marker; P: positive control; N: negative control; C: control line; T: test line.

The extracted DNA of *O. sinensis* with a continuous dilution (0.7–179.2 ng/μL) was prepared with ddH_2_O to evaluate the sensitivity of RPA-NAH-LFS. RPA was performed using various concentrations of DNA as templates to determine the limit of detection (LOD) of the RPA-NAH-LFS assay. As expressed in Figure 9A, analysis of PCR amplicons using AGE, the intensity of AGE bands gradually diminishes as the concentration of *O. sinensis* DNA is decreased from 179.2 ng/μL to 2.8 ng/μL. When the DNA concentration falls below 2.8 ng/μL, no distinct bands of a specific size are visible on the gel, suggesting that the LOD of the PCR-AGE for *O. sinensis* DNA is 2.8 ng/μL. Further analysis using RPA-NAH-LFS, as shown in Figure 9B, revealed that the T line gradually fades as the concentration of *O. sinensis* DNA decreases. When the concentration reaches 0.7 ng/μL, the T line on NAH-LFS does not exhibit any color. At the template concentration of 1.4 ng/μL, there is a visibly significant difference in color intensity from the negative control. Therefore, the detection sensitivity of the RPA-NAH-LFS for *O. sinensis* DNA is 1.4 ng/μL. The sensitivity of RPA-NAH-LFS is two times lower than that of the PCR-AGE.

### 2.7. Detection Commercial O. sinensis Samples by RPA-NAH-LFS

**Figure 10:**
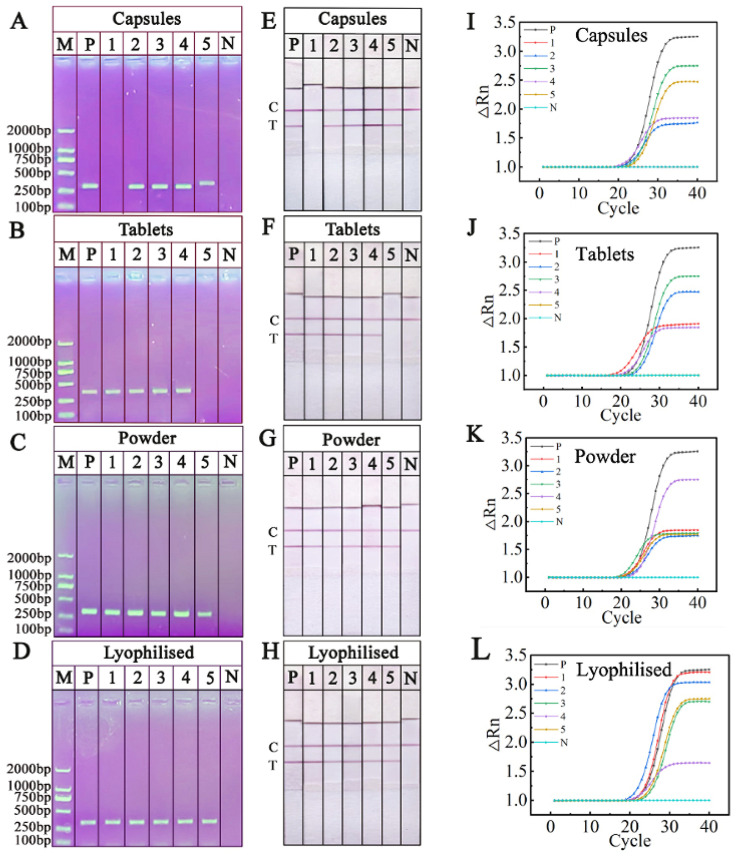
Testing results of PCR-AGE (**A**–**D**), RPA-NAH-LFS (**G**,**H**), and RT-PCR (**I**–**L**) for commercial *O. sinensis* samples. (**A**,**E**,**I**) *O. sinensis* capsule products; (**B**,**F**,**J**) *O. sinensis* tablet products; (**C**,**G**,**K**) *O. sinensis* powder products; (**D**,**H**,**L**) lyophilized *O. sinensis* products. P: positive control; N: negative control; C: control line; T: test line.

In order to evaluate the effectiveness of RPA-NAH-LFS in analyzing commercial *O. sinensis* samples, 20 products labeled as authentic *O. sinensis* were collected and tested under optimized conditions. The results, illustrated in Figure 10, revealed that one out of five *O. sinensis* capsule products showed only a C line, indicating adulteration. One *O. sinensis* tablets products was found to lack genuine *O. sinensis* ingredients. Among the five *O. sinensis* powder products and five lyophilized *O. sinensis* products, displayed a distinct red color on the T line, indicating their authenticity. The results obtained from RPA-NAH-LFS (Figure 10A–D) was identical with PCR-AGE (Figure 10E–H) and RT-PCR (Figure 10I–L).

## 3. Discussion

*O. sinensis* a revered and rare edible fungus, is considered as superior tonic and herbal remedy, often compared to ginseng in terms of its benefits and commanding a higher price. The medicinal properties of *O. sinensis* include antioxidant effect, fatigue relief, anti-tumor potential, increased vitality, and cardiovascular benefits [27]. With the significant increase in price and demand for *O. sinensis*, other related fungi with lower mass have emerged as potential substitutes [28]. Reports indicate that while the annual production of authentic *O. sinensis* is around 185 tons, total market sales exceed 200 tons both domestically and internationally [29]. This disparity raises concerns about market disruptions, diminished consumer trust, and potential health risks associated with counterfeit products. Given these challenges, there is an urgent need for a reliable, point-of-care method to distinguish authentic *O. sinensis* from counterfeit products to protect consumers and ensure product authenticity.

DNA-based detection technology is increasingly being recognized for its precision and practicality in authenticating Chinese medicinal materials. In this study, wild *O. sinensis* within commercial products was identified using RPA coupled with NAH-LFS. Furthermore, the accuracy of the RPA-NAH-LFS results was confirmed through PCR-AGE analysis. The results indicated that RPA-NAH-LFS can accurately identify *O. sinensis* from counterfeit samples. The successful amplification and visualization of a 1.4 ng/µL DNA template using RPA-NAH-LFS suggest that its sensitivity is higher than that of PCR-AGE. Moreover, the entire RPA-NAH-LFS process can be completed in just 16 min (1 min for DNA extraction, 10 min for RPA, and 5 min for NAH-LFS). As shown in Table 3, this RPA-NAH-LFS demonstrates a greater speed, simplicity, and cost-effectiveness compared to PCR-AGE and other alternative methods [9,30]. Importantly, specificity testing confirmed no cross-reactivity with five other fungi, and the method is user-friendly for non-expert operators, requiring only a digital water bath and featuring straightforward operational steps.

In previous studies, various molecular biology methods have been utilized for the identification of *O. sinensis* and related products. For instance, Li et al. designed species-specific primers for PCR amplification of internal transcribed spacer sequences, with the LOD of 8 ng target DNA in 25 µL reaction volume [31]. Thanh et al. employed purpose-made capillary electrophoresis-based technology to determine the nucleosides of *O. sinensis*, achieving an LOD range of 11.2–22.0 μg/g [32]. Duan et al. introduced a multispectral imaging technique to enhance the efficiency of *O. sinensis* detection, achieving an impressive accuracy of 96.3% [33]. However, the existing studies have often faced challenges such as high equipment requirements, time-consuming procedures, the need for specialized expertise, and reliance on multiple statistical models and algorithms. The RPA-NAH-LFS method established in this study overcomes these limitations effectively.

AuNPs are prepared by the citrate reduction method and stabilized by interaction forces between citrate ions. Even micro-concentrations of NaCl (50 mM) will cause the AuNPs to aggregate, resulting in the solution becoming colorless [34]. However, when SH probes are adsorbed on the surface of AuNPs, the stability of AuNPs is significantly improved, even with the addition of 300 mM NaCl (Figure 3B). In this study, the solution remained burgundy after adding 300 mM NaCl, indicating that no aggregation of AuNPs occurred and proving that the SH probe was successfully coupled to the AuNP surface.

The NAH-LFS based on nucleic acid hybridization to catch target DNA in this study is ideal for rapid on-site testing and does not rely on costly antibodies. The stability of the NAH-LFS can be maintained for up to one year by ensuring dry storage conditions during transportation. The RPA-NAH-LFS method developed in this study has broader applications beyond *O. sinensis* authentication; it can be adapted to identify other traditional Chinese medicines by substituting the specific primer set. Liu et al. (2018) successfully utilized RPA-LFS to authenticate *Ginkgo folium* products, demonstrating superior accuracy compared to PCR-based methods [35]. Zhao et al. (2019) applied the RPA in combination with immunoassay LFS method to distinguish saffron from its adulterants, showcasing its potential versatility in analyzing processed products [36]. Meanwhile, the RPA-NAH-LFS method has been extensively employed in various fields, including meat product identification [37], virus detection [38], identification of pathogenic bacteria [39], and recognition of valuable botanical [40]. Compared to previous studies [37,41], the innovation lies in the fact that the entire *O. sinensis* detection process, from DNA extraction to the visualization of test results, does not require instruments or complex operations, and can be completed within 16 min. Additionally, there are limited studies in the literature on the utilization of RPA-NAH-LFS for *O. sinensis* identification. In summary, the RPA-NAH-LFS method enables on-site detection with limited resources across diverse areas to ensure food safety and prevent intentional contamination.

## 4. Materials and Methods

### 4.1. Materials

Chloroauric acid tetrahydrate (HAuCl_4_·4H_2_O), sodium chloride, agarose, sodium citrate, sodium dihydrogen phosphate, disodium hydrogen phosphate, hydrochloric acid, and tris (2-carboxyethyl) phenol hydrochloride (TCEP), were purchased from Aladdin Reagent (Shanghai, China). Streptomycin avidin, 2-[4-(2-hydroxyethyl)-1-piperazinyl] ethanesulfonic acid (HEPES), sucrose, Tween-20, and albumin bovine V (BSA) were obtained from Solebao Technology (Beijing, China). RPA basic amplification kit was obtained from TwistDxInc (Cambridge, UK). The Tiangen polysaccharide polyphenol plant genome DNA extraction kit was obtained from Tiangen Biotechnology (Beijing, China). Nitrocellulose (NC) membrane and glass fiber obtained from Jinbiao Biotechnology Co., Ltd. (Shanghai, China). The probes and primers were purchased from GENEWIZ (Suzhou, China). The 2×Taq PCR Mix plus were purchased from LabEAD (Beijing, China). Goldview nucleic acid dye was purchased from Biotopped (Beijing, China).

### 4.2. Sample Preparation and DNA Extraction

A total of six samples were purchased from various medicinal material markets in Tangshan, including *O. sinensis*, *C. gunnii*, *C. cicadae*, *C. militaris*, *O. nutans*, and *yungui Cordyceps*. These samples were visually identified by Beijing Tong Ren Tang before the experiment. Additionally, 20 commercial products of *O. sinensis* were purchased from online supermarket platforms and pharmacies; detailed information is provided in Table 4. All samples were dried for 24 h under a vacuum at 60 °C, and then ground into powder individually.

In this study, we compared the following two DNA extraction methods: (1) DNA extraction of all samples (0.02 g each) using a commercial kit (DP360, TIANGEN, Beijing, China) following the specified protocol; and (2) DNA extraction using tungsten with slight modifications [42,43]. Polymethyl methacrylate (PMMA), which features three holes, was utilized as a support for the fabrication of three tungsten needles. Each needle had a tip measuring 4 mm and a handle measuring 7 mm. After the three tungsten needles were affixed to the PMMA, a handle was attached to the opposite side of the PMMA (Figure 1). Specifically, approximately 100 μL of ddH_2_O was added to 0.02 g of *O. sinensis* powder and the needles were immersed in the solution for 1 min. The DNA was absorbed onto the surface of the needles, which were then washed with 100 μL of ddH_2_O. The extracted DNA was quantified using an ultra-micro-spectrophotometer K5800 (KAIAO, Beijing, China) by measuring the absorbance at A260/280 nm and A260/230. Subsequently, the extracted DNA from tungsten needles was subjected to PCR amplification and compared with the results obtained with commercial kits. The extracted DNA was diluted and stored at −20 °C until further use.

### 4.3. Synthesis of AuNPs and Conjunction with SH-Probes

AuNPs were synthesized following established protocols [41,44] to obtain magenta spherical AuNPs with an average diameter of 20 nm using the citrate reduction of HAuCl_4_. Briefly, 0.5 mL 1% HAuCl_4_ was added into a conical flask containing 49 mL of ultrapure water. The solution was stirred and heated to boiling. Subsequently, 1.125 mL 1% trisodium citrate was injected to the flask while continuing to heat and stir until it turned magenta, indicating that the AuNPs were obtained. For the combination of AuNPs and SH-probes, the following steps were carried out:

1. SH probes (10 μL 100 μM) were first reduced by adding them into TCEP (10 μL, 1 mM) and adjusted the solution pH to 3.0 with citrate–HCl buffer. The mixture was then incubated at room temperature for 1 h.
2. A total of 1330 μL AuNPs was injected into the above SH probe solution. After 10 min of reaction, citrate-HCl (30 μL, 10 mM) was added to AuNPs-SH probe solution to adjust the pH to 3.0. Then, NaCl (10 μL, 2.0 M) was added sequentially and allowed to react for additional 10 min.
3. The pH of the AuNPs-SH probe solution was adjusted to 7.5 by HEPES buffer (100 μL, 500 mM).
4. The AuNPs-SH probes were incubated for 1 h before being centrifuged at 2000× *g* for 10 min. The supernatant was removed, and the pellet was resuspended in 90 μL buffer (1 mM Tris-HCl, 10% sucrose, 0.5% Tween-20, and 1% BSA, pH of 7.6). The AuNPs-SH probe were sprayed onto the conjugate pad at 20 μL/cm.

### 4.4. Design of PCR/RPA Primers and NAH-LFS Probes

For the amplification of the *O. sinensis* DNA (RefSeq: KF696589.1), four primers were designed based on the specification of TwistAmp Basic kits. These primers were designed using Primer Premier 6.0 software and subsequently screened for accuracy through BLAST at NCBI and RPA amplification. The primer that produced the specific and brightest band on the AGE was selected as the optimum primer and used in the subsequent RPA-NAH-LFS.

In addition, three probes involved on NAH-LFS were as follows:

1. SH probes on AuNPs, which are reverse complementary to the tag sequence of forward primers;
2. biotin-labeled capture probe, which are reverse complementary to tag sequence of reverse primers; and
3. biotin-labeled control probe, which complementarily hybridize with SH probes.

The RPA amplicons have two single-strand tails (F-tag and R-tag) at the two opposite ends, which can bind to the reverse complementary capture probes at the T line of NAH-LFS and SH-AuNPs, respectively. Meanwhile, control probes were fixed on the control line (C line), which were reverse complementary to SH-AuNPs. The structure of the primer set is presented in Figure 1 and Table 2.

### 4.5. PCR and AGE Testing

Each PCR reaction included 10 μL 2×Taq PCR Mix plus, 1 μL 10 μM forward/reverse primers, 7 μL ddH_2_O, and 1 μL template DNA of *O. sinensis*. PCR was accomplished in a PCR instrument (Eppendorf, Germany) with the following conditions: a preheating step at 95 °C for 3 min, 30 cycles of 95 °C for 1 min, 54 °C for 40 s, 72 °C for 50 s, final extension at 72 °C for 5 min. The amplicons were detected by 1% AGE with 1 μL goldview nucleic acid dye.

### 4.6. Assembly and Application of the NAH-LFS

The NAH-LFS assembly involved attaching the sample pad, absorbing pad, NC membrane, and conjugate pad to the supporting backplane. To prepare the NC membrane, a complex consisting of biotinylated capture/control probes (5 μL, 10 μM) and streptavidin (2.5 μL, 1 mg/mL) was mixed with 11.6 μL deionized water and then sprayed on the T line/C line. The interaction between biotin and streptavidin enables the immobilization of probes on the NC membrane. The T line was positioned 1.5 cm from the top margin of the NC membrane and the C line was located at 1 cm from the top margin of the NC membrane. Both the NC membrane and conjugate pad were vacuum-dried at 38 °C for 4 h. Eventually, the NAH-LFS was assembled sequentially, and overlapped between each parts by 2 mm to ensure smooth capillary forces; then, the LFS was cut into 4 mm section using a cutter (Jinbiao Biotechnology Co., Ltd., Shanghai, China). The sample mixture was added to the sample pad, allowing it to migrate along the NAH-LFS. When the sample flows to the conjugate pad, the tag sequence of the forward primer was captured by SH-AuNPs and resulted in the RPA amplicons being labeled with AuNPs. Subsequently, the RPA amplicons–AuNPs continue to flow to the test line (T line), where the R-tag hybridized with capture probe preloaded on the T line, leading to the formation of a red line due to the colorimetric effect of the AuNPs. Finally, excessive SH-AuNPs diffuse to the C line, which were captured by control probes to form another red band indicating that the NAH-LFS is working properly. The working principle of NAH-LFS is shown in Figure 1.

### 4.7. Optimizing RPA Conditions and NAH-LFS Working Conditions

#### 4.7.1. Optimizing RPA Conditions

The optimization of RPA conditions is essential for ensuring efficient amplification of the target DNA. Each RPA amplification reaction was carried out in a 50 μL reaction mixture containing primers, ddH_2_O, MgAcO, rehydration buffer, and lysozyme. Following amplification, the RPA products were detected using 1% AGE. The following parameters were optimized using the TwistAmp Basic Kit:

1. Reaction temperature and incubation time: RPA amplification reactions were conducted at temperatures ranging from 37 to 41 °C and varying incubation times between 5 and 20 min. These parameters were systematically varied to determine the optimal conditions for efficient amplification.
2. Primer concentration: The concentration of the primers in the RPA reaction mixture was optimized within the range of 1–1.6 μL (10 μM) to determine the ideal amount that promotes robust amplification.
3. Magnesium Acetate (MgAcO) concentration: The MgAcO concentration in the reaction mixture was varied from 2 μL (280 mM) to 3.5 μL (280 mM) to identify the optimal concentration that facilitates efficient amplification.

This comprehensive approach allowed for the confirmation of efficient amplification and successful detection of the target DNA.

#### 4.7.2. Optimizing NAH-LFS Working Conditions

For improving performance of NAH-LFS, different types of WS and NC membrane with different pore size were compared to determine the optimal conditions. In the NAH-LFS phase, the RPA amplicon was diluted 1:10 with four types of WS (WS1: 0.01 M PBS, pH of 7.0; WS2: 0.01 M PBS with 0.05% Tween-20 and 1% BSA, pH of 7.0; WS3: 0.01M PBS, pH of 8.0; WS4: 0.01 M PBS, 0.05% Tween-20 and 1% BSA, pH of 8.0). Subsequently, 100 μL of the diluted solution was dropped onto the sample pad of NAH-LFS, and the result was observed within 5 min.

The flow rate (FR) and pore diameter of NC membranes play crucial roles in the performance of NAH-LFS. Therefore, three types of NC membranes with varying FR (FR = 33.75, 45.00, 22.50 cm/s) were evaluated to select the optimal NC membrane and working solution combination.

The WS and the NC membrane that produced the clearest red bands were selected as the suitable NAH-LFS working conditions. Positive results were indicated by the appearance of visible C and T lines on the NAH-LFS, while negative results showed only the C line. Other forms of coloration were invalid for the NAH-LFS assay.

### 4.8. Specificity and Sensitivity of the RPA-NAH-LFS Assay

To assess the specificity of the RPA-NAH-LFS assay, five non-target fungal species, namely *C. gunnii*; *C. cicadae*; *C. militaris*; *O. nutans;* and *yungui Cordyceps*, were selected. The genomic DNA of these non-target fungal species was used as templates for RPA reaction, and the resulting RPA products were examined by both NAH-LFS and AGE to confirm specificity. To determine the sensitivity of the RPA-NAH-LFS, the genomic DNA of *O. sinensis* was serially diluted with ddH_2_O to generate RPA templates. The resulting RPA products were then examined using both NAH-LFS and AGE to determine the limit of detection (LOD) of the assay. During the RPA amplification, deionized water served as a negative control to ensure the accuracy of the amplification process. The specificity and sensitivity of the RPA-NAH-LFS were evaluated through triplicate determinations to confirm repeatability and reliability of the results.

### 4.9. Detection of Commercial O. sinensis Products

To validate the practical application of the RPA-NAH-LFS assay under optimal conditions, 20 commercial *O. sinensis* products were detected by RPA-NAH-LFS assay. The products, including 5 *O. sinensis* capsules, 5 *O. sinensis* tablets, 5 *O. sinensis* powder, 5 lyophilized *O. sinensis*, were purchased from online supermarket platforms and pharmacies. The DNA of each product was extracted, followed by RPA amplification and NAH-LFS detection.

For the detection of commercial *O. sinensis* products, DNA was extracted with tungsten and screened-specific primers were employed for RPA amplification. After amplification, 10 μL of the RPA products were mixed with 90 μL of the WS in a centrifuge tube. And the mixture was then applied to the sample pad of the NAH-LFS and allowed to migrate along the strip. Results were visible within 5 min, providing a rapid and on-site detection for the presence of *O. sinensis* in the commercial products. The results were verified by PCR-AGE.

## 5. Conclusions

In this study, a visual RPA-NAH-LFS for the direct, rapid, and in situ detection of *O. sinensis* was established, utilizing AuNPs as labels to generate a distinctive red color signal. The detection process can be completed within 27 min using only a digital water bath. By incorporating spacer9 and a tag sequence into the RPA primer set for detecting *O. sinensis* DNA, the LFS relying on nucleic acid hybridization were established. The effectiveness of this RPA-NAH-LFS confirms the absence of nonspecific reactions between *O. sinensis* and five other fungi. The RPA-NAH-LFS can efficiently detects *O. sinensis* DNA concentrations as low as 1.4 ng/μL which is twice lower than PCR-AGE. In commercial product inspections, the results from RPA-NAH-LFS and PCR-AGE were consistent, revealing that the RPA-NAH-LFS is robust and accurate in detecting *O. sinensis*. Based on the advantages of time-saving, simplicity, and high levels of accuracy, specificity, and sensitivity, the RPA-NAH-LFS as a point-of-care detection method is expected to be widely adopted for authentic *O. sinensis* identification. Importantly, it also can be used to identify other forms of counterfeiting by substituting the specific primer set, indicating its potential for widespread use in vitro diagnostics.

## Figures and Tables

**Table 1:** Comparison of DNA extraction by commercial kit and tungsten (*n* = 3).

Method	A260/230	A260/280	A260	DNA Concentration	Time	Installation
Tungsten	0.518 ± 0.03	1.323 ± 0.13	1.004	50.2 ± 1.27 ng/µL	1 min	/
Commercial kit	0.683 ± 0.06	1.741 ± 0.16	1.430	71.5 ± 2.59 ng/µL	50 min	Water bath, Centrifuge, Vortex oscillator

**Table 2:** Primers and probes needed by RPA-NAH-LFS.

Name	Sequences (5′→3′)	Tag Sequence (5′→3′)	Function
Primer 1	TTGTAGAAAACGGGGCAGGA	/	PCR
	CCGCAGGGTCGAAAAATGAAG	/	
Primer 2	TAGAAAACGGGGCAGGAACA	ATTTTTCACTGGGTTTATAGT-spacer9	PCR/RPA
	TTGTAGAAAACGGGGCAGGA	TCGAGTGACAGCTAATGTGTGATT-spacer9	
Primer 3	TTGGTGAACCAGCGGAGGGATCATT	/	PCR
	GCTTGCTTCTTGACTGAGAGGTGCC	/	
Primer 4	ATTAAGTCGTGGAAATG	/	PCR
	GATCAGGAATAGTGGGA	/	
SH-probe	ACTATAAACCCAGTGAAAAAT	-SH	NAH-LFS
Control probe	ATTTTTCAGGGTTTTATAGT	Biotin	NAH-LFS
Capture probe	AATCACACATTAGCTGTCACTCGA	Biotin	NAH-LFS

**Table 3:** Comparison of RPA-NAH-LFS with other techniques.

Method	Time		Cost	
	Amplification	Detection	Equipment	Reagent
PCR-AGE	120 min	45 min	Thermal cycling equipment	1.00 $
PCR-ICTS	120 min	5–15 min	Thermal cycling equipment	1.50 $
RPA-ICTS	10 min	5 min	Water bath	6.92 $
RPA-NAH-LFS	10 min	5 min	Water bath	6.42 $

ICTS: immunochromatographic test strip.

**Table 4:** Detailed information and test results of 20 commercial *O. sinensis* samples.

No.	Sample	From	Test Results ^a^
1	*O. sinensis* capsule 1	China Beijing Tang	+
2	*O. sinensis* capsule 2	Mannings	−
3	*O. sinensis* capsule 3	Vitahealth	+
4	*O. sinensis* capsule 4	Alibaba Health	+
5	*O. sinensis* capsule 5	Wei shiya	+
6	*O. sinensis* tablet 1	China Beijing Tang	+
7	*O. sinensis* tablet 2	Mannings	+
8	*O. sinensis* tablet 3	Vitahealth	+
9	*O. sinensis* tablet 4	Alibaba Health	+
10	*O. sinensis* tablet 5	Wei shiya	−
11	*O. sinensis* powder 1	China Beijing Tang	+
12	*O. sinensis* powder 2	Mannings	+
13	*O. sinensis* powder 3	Vitahealth	+
14	*O. sinensis* powder 4	Alibaba Health	+
15	*O. sinensis* powder 5	Wei shiya	+
16	lyophilized *O. sinensis* 1	China Beijing Tang	+
17	lyophilized *O. sinensis* 2	Mannings	+
18	lyophilized *O. sinensis* 3	Vitahealth	+
19	lyophilized *O. sinensis* 4	Alibaba Health	+
20	lyophilized *O. sinensis* 5	Wei shiya	+

^a^ (+) positive result; (−) negative result.

## Data Availability

All data were included in the manuscript.

## Supplementary Materials

The following supporting information can be downloaded at: https://www.mdpi.com/article/10.3390/ijms252413599/s1.
