# Supplementary material for: Visualized Nucleic Acid Hybridization Lateral Flow Strip Integrating with Microneedle for the Point-of-Care Authentication of Ophiocordyceps sinensis
Source: Int J Mol Sci. 2024 Dec 19;25(24):13599. doi: 10.3390/ijms252413599 (PMC11677120; doi:10.3390/ijms252413599)
Supplement: Supplementary file 1 [file ijms-25-13599-s001.zip › ijms-3311431-supplementary.pdf]

Supporting Information for:

Visualizated nucleic acid hybirdization lateral flow strip integrating with microneedle for the point-of-care authentication of Ophiocordyceps sinensis

Haibin Liu[1], Xinyue Wang[1], Hang Tian[1], Yi Yuan[1], Jing Wang[1], Yani Cheng[1], Linyao Sun[1], Hongshuo Chen[2*], Xiaoming Song[1*]

1 College of life sciences, North China University of Science and Technology, Tangshan, Hebei, 063200, China; liuhaibinhappy1013@126.com

2 College of Electrical Engineering, North China University of Science and Technology, Tangshan, Hebei, 063200, China; chs@ncst.edu.cn

[*] Corresponding author

* Correspondence: chs@ncst.edu.cn; Tel.: +86 18322596403

songxm@ncst.edu.cn; Tel.: +86 18332752519

Tel.: +86 18322596403

E-mail address: songxm@ncst.edu.cn

Supplementary figure

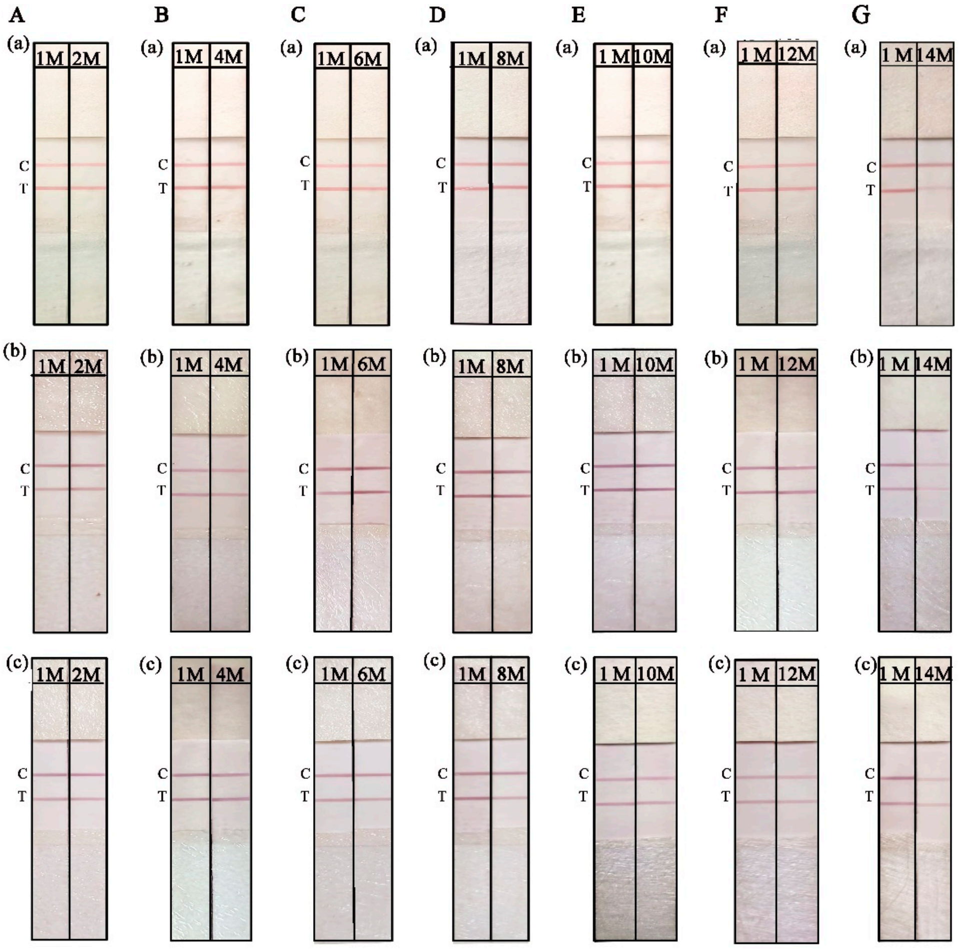

Figure S1 The retention period of the NAH-LFS was tested in triplicate. 1 M, 2 M, 4 M, 6 M, 8 M, 10 M means NAH-LFS stored for 1 month, 2 months, 4 months, 6 months, 8 months, 10 months, 12 months, 14 months, respectively. C: control line, T: test line.
